# The Metabolic Signatures of Surviving Cotwins in Cases of Single Intrauterine Fetal Death During Monochorionic Diamniotic Pregnancy: A Prospective Case-Control Study

**DOI:** 10.3389/fmolb.2022.799902

**Published:** 2022-04-08

**Authors:** Xiyao Liu, Huijia Fu, Li Wen, Fangyu Zhu, Yue Wu, Zhi Chen, Richard Saffery, Chang Chen, Hongbo Qi, Chao Tong, Philip N. Baker, Mark D. Kilby

**Affiliations:** ^1^ State Key Laboratory of Maternal and Fetal Medicine of Chongqing Municipality, Chongqing Medical University, Chongqing, China; ^2^ International Collaborative Laboratory of Reproduction and Development of Chinese Ministry of Education, Chongqing Medical University, Chongqing, China; ^3^ Department of Reproductive Medicine, The First Affiliated Hospital of Chongqing Medical University, Chongqing, China; ^4^ Molecular Immunity, Murdoch Children’s Research Institute, Parkville, VIC, Australia; ^5^ Institute of Life Sciences, Chongqing Medical University, Chongqing, China; ^6^ Chongqing Women and Children’s Health Center, Chongqing, China; ^7^ College of Life Sciences, University of Leicester, Leicester, United Kingdom; ^8^ College of Medical and Dental Sciences, University of Birmingham, Birmingham, United Kingdom; ^9^ Fetal Medicine Centre, Birmingham Women’s and Children’s Foundation Trust, Birmingham, United Kingdom

**Keywords:** LC-MS, maternal-fetal interface, metabolites, metabolome, radiofrequency ablation, spontaneous fetal death

## Abstract

**Introduction:** Single intrauterine fetal death (sIUFD) in monochorionic diamniotic (MCDA) twin pregnancy may be associated with adverse clinical outcomes and possible metabolic changes in the surviving co-twin. Metabolomic profiling has not been undertaken before in these complex twin pregnancies.

**Methods:** In this prospectively collected case-control study, three cross-cohort comparisons were made between sIUFD MCDA (*n* = 16), uncomplicated MCDA (*n* = 16, eight pairs), and uncomplicated singleton pregnancies (*n* = 8). To identify major sources of variation within the sIUFD MCDA cohort, a secondary comparison was conducted between spontaneous sIUFD (*n* = 8) and sIUFD in MCDA twins due to selective termination of a single abnormal fetus by radiofrequency ablation (RFA) (*n* = 8). Metabolomics analysis of placental tissue and umbilical cord plasma was performed using LC-MS profiling. The underlying metabolic networks and pathways were analyzed by web-based platforms. Associations and statistical correlations of all identified differential metabolites with neonatal birthweight and birth length were assessed by multivariable linear regression, adjusted for maternal age and gestation.

**Results:** Across four comparisons, 131 and 111 differential metabolites were identified in placental tissue and cord plasma, respectively, with the highest variation seen between the spontaneous vs. single-induced IUFD in MCDA twins by RFA in the cord plasma. Conversely, the number of viable fetuses and the presence of sIUFD in MCDA twins had the highest impact on metabolite variation in placental tissue. Compounds correlated with fetal growth including placental acylcarnitines and gangliosides, along with specific amino acids (e.g., histidinyl-hydroxyproline), xenobiotics and biliverdin in cord plasma.

**Conclusion:** sIUFD in MCDA twin pregnancy correlates with distinctive metabolic signatures, mostly in fatty acyls and complex lipids, in placental tissue and cord plasma of the surviving cotwin. Some metabolites are also associated with fetal growth.

## Introduction

The prevalence of multiple pregnancies is rising internationally due to the increasing use of assisted reproductive technology (ART) and the trend toward advanced maternal age in pregnancy (Umstad et al., 2013). The majority of multiple pregnancies are twin pregnancies (98%) (ACOG Practice Bulletin No, 2014) and compared with singleton pregnancies, these pregnancies have a higher risk of fetal and neonatal morbidity and mortality (National Institute for Health and Clinical Excellence, 2011; ACOG Practice Bulletin No, 2014). The majority of twin pregnancies (∼80%) are dichorionic diamniotic, with each developing fetus attached to its own placenta within a separate amniotic sac. The remaining 20% of twins share a single placenta (monochorionic; MC), which is associated with increased fetal mortality and morbidity predominantly due to conjoining of the fetal circulations by the presence of placental vascular anastomoses (Hack et al., 2008; Southwest Thames Obstetric Research Collaborative, 2012).

Single intrauterine fetal death (sIUFD) complicates up to 6.8% of all twin pregnancies but is more common in MC twin pregnancies (7.5%) (Morris et al., 2020). sIUFD may occur spontaneously (27% of sIUFDs) or associated with a single fetus termination (often using radiofrequency ablation (RFA)) as part of clinical management when there is discordant fetal anomalies or severe selective growth restriction (Morris et al., 2020). The surviving twin of a sIUFD MCDA twin pregnancy, particularly if single twin demise occurs spontaneously, has a higher risk of fetal mortality, associated neurologic injury (secondary to cerebral ischemia), and preterm birth (PTB) (Santema et al., 1995; Hillman et al., 2011; Mackie et al., 2019). It is the angioarchitecture of the intertwin placental anastomoses that dictate the risk of ischemic injury and in iatrogenic sIUFD the techniques used (of radiofrequency ablation) attempts to reduce the risk of any intertwin acute inter-twin transfusion through the placental anastomoses.

As with other prenatal complications (Wang et al., 2018), sIUFD may lead to a suboptimal intrauterine environment and metabolic changes in the developing fetus, which can negatively impact the pregnancy outcome. Even so, metabolomic profiling has not previously been conducted in sIUFD cases relative to other uncomplicated pregnancies. Furthermore, the impact of spontaneously occurring *versus* induced (by selective termination of pregnancy) sIUFD in MCDA twins on the metabolic profile of the surviving twin has yet to be assessed.

In this prospective case-cohort control study, we performed a metabolomics analysis of placental tissue and cord plasma from surviving cotwins in monochorionic diamniotic (MCDA) pregnancies complicated by spontaneous or induced sIUFD in MCDA twins relative to uncomplicated MCDA twins and singleton pregnancies. The underlying associations between the identified differential metabolites and neonatal birthweight and birth length were also investigated.

## Methods

### Participants

From the beginning of 2016 to the end of 2018, participants were prospectively recruited in the Department of Obstetrics and Gynecology at The First Affiliated Hospital of Chongqing Medical University.

**FIGURE 1:**
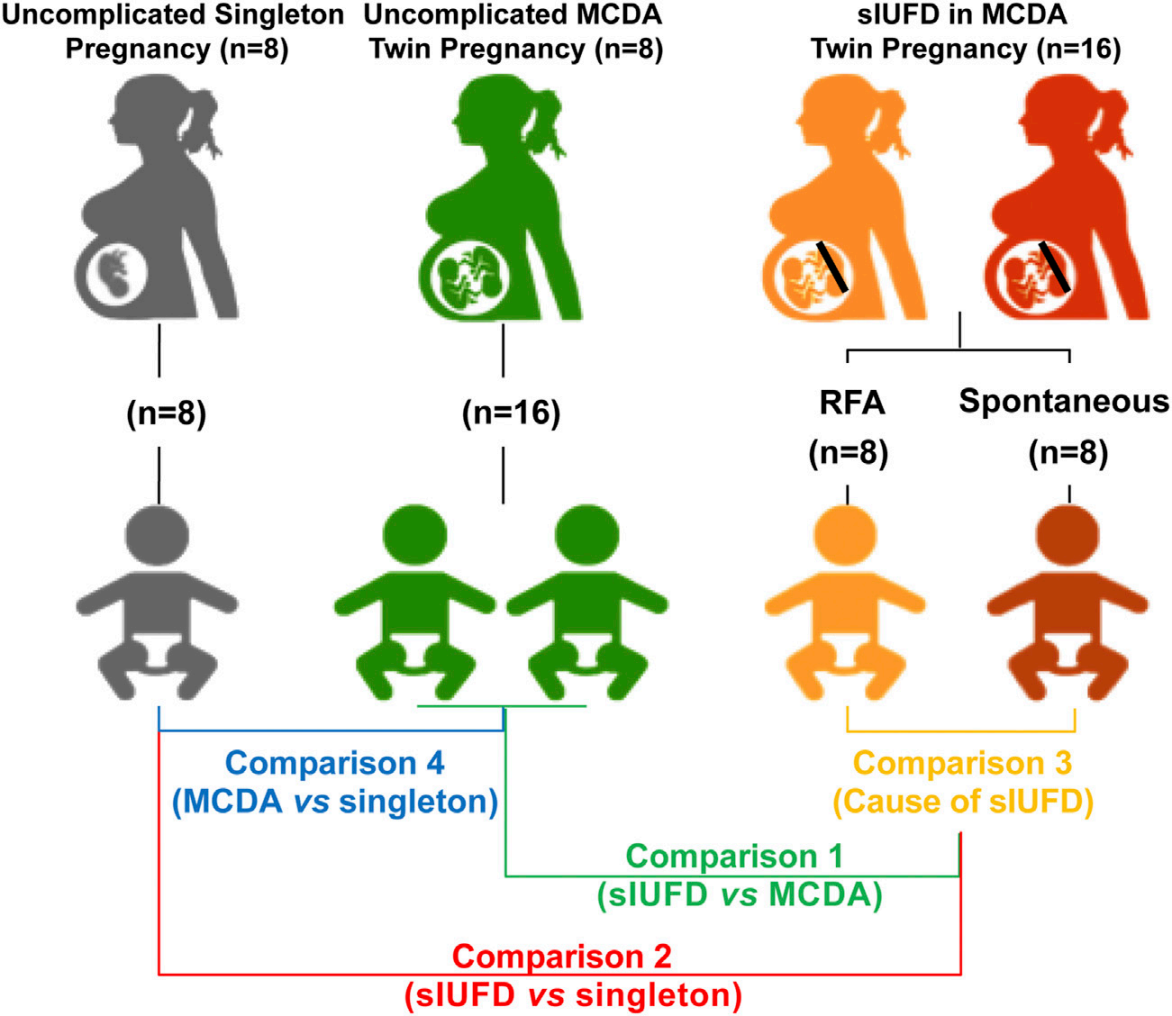
Schematic diagram of study design. The study cohorts (with their representative colors) are as follows: sixteen sIUFD MCDA cases, namely, eight spontaneous sIUFD cases (red) and eight post-RFA sIUFD cases (orange), were included; eight maternal age-matched uncomplicated MCDA twin pregnancies (16 neonates, green) and eight age-matched uncomplicated singleton pregnancies (grey) were also selected. The four comparisons (with corresponding iconic colors) are C1 (green): (RFA + Spontaneous) vs. MCDA; C2 (red): (RFA + Spontaneous) vs. Singleton; C3 (orange): Spontaneous vs. RFA; C4 (blue): MCDA vs. Singleton. Abbreviations: MCDA, monochorionic diamniotic; sIUFD, single intrauterine fetal death; RFA, radiofrequency ablation.

Participants included: 1) sixteen cases of MCDA twin pregnancies complicated by sIUFD, among which eight were spontaneous sIUFDs and the other eight were patients who had undergone radiofrequency ablation (Lee et al., 2007; Kumar et al., 2014); 2) eight cases of maternal age-matched uncomplicated MCDA twin pregnancies, and 3) eight cases of age-matched uncomplicated singleton pregnancies (Figure 1).

MCDA twin pregnancies, were diagnosed as monochorionic and diamniotic, using ultrasound with the follow features: 1) a single gestational sac and placenta by 10 weeks of gestation and 2) T-sign of the intertwin membrane and absent twin peak (lambda) sign, with a single placenta between 11 and 14 gestational weeks. The placental features of MCDA twin pregnancy were further confirmed by postpartum clinical examination (Kristiansen et al., 2015).

Spontaneous sIUFD in MCDA twin pregnancy was diagnosed by ultrasound after clinical presentation. MCDA twin pregnancy with sIUFD following RFA (due to a discordant fetal anomaly) was also confirmed by ultrasonography postoperatively.

A conservative strategy was followed after sIUFD in MCDA twins diagnosis unless the pregnancy was >34 weeks and then delivery was considered with care being individualized (Jelin et al., 2010). Throughout conservative management, the antenatal progress of mothers and fetuses was monitored by ultrasound at regular intervals. Cases were not included if 1) intrauterine death of the cotwin was diagnosed simultaneously with sIUFD or during the expectant treatment; or 2) if sIUFD was first diagnosed at delivery.

The clinical characteristics of these participants were recorded within 24 h after delivery, which included: 1) maternal age, body mass index [BMI (before pregnancy, at delivery, and overall increment)], lifestyle behavior (smoking and alcohol use), parity, and mode of conception (ART or spontaneous); 2) obstetric complications; 3) method of and gestational age (GA) at delivery; and 4) neonatal sex, birthweight, birth length, Apgar scores (1, 5, and 10 min), and NICU admission. For patients in the sIUFD group, information regarding the etiology and GA of sIUFD was added. The interval between sIUFD occurrence and pregnancy termination was calculated as well. The study was approved by the Ethics Committee of Chongqing Medical University (No. 201530). Written informed consent was obtained from all participants.

### Sample Collection

As previously described (Wang et al., 2018), placental tissues and cord plasma samples were obtained from participants immediately after delivery. The sampled region of the placenta was on the maternal side, adjacent to the insertion point for the umbilical cord of the living baby (or the placental edge nearest to the umbilical cord insertion in a velamentous inserted cord). After collection, the placental tissue was immediately washed with precooled 0.9% sterile normal saline three times to remove blood clots, dissected into pieces of approximately 5 mm^3^, and then blotted dry. The dry tissue was rapidly frozen in liquid nitrogen and stored at −80°C. A total of 4 ml cord blood was collected from the umbilical vein of the living fetuses into an EDTA-coated blood collection tube, which was then centrifuged at 3,000 rpm for 15 min at 4°C. The supernatant plasma was aliquoted into new sterile tubes and stored at −80°C.

### Metabolite Extraction

This is the standard protocol used in our laboratory (Chen et al., 2018; Chen et al., 2020). The placental tissue (prepared as stated) was blotted dry and weighed to 50 ± 0.5 mg in a fresh tube, and then 50 μl precooled saline and five repeated 10-s sonication cycles were applied to generate the placental homogenate for use. The cord plasma was thawed on ice and vortexed, and 50 μl of the homogeneous plasma was transferred into a new tube for use. To extract metabolites, each homogenized sample was vortex-mixed with 300 μl methanol (for placental tissue) or 500 μl acetonitrile (for cord plasma). After centrifugation at 14,000 rpm for 15 min at 4°C, the supernatant was transferred to a new tube, vacuum-dried using a CentriVap^®^ concentrator (Labconco, Kansas City, MO, United States) at 30°C, and cryopreserved at −80°C. Before further determination, the dried extract was reconstituted in 100 μl 50% acetonitrile and centrifuged at 14,000 rpm for 5 min at 4°C. Finally, 80 μl of the supernatant was transferred to a fresh vial specialized for LC-MS profiling. The quality control (QC) sample was prepared, separately for placenta tissue and cord plasma, by combining 5 μl aliquots from each reconstituted sample of corresponding specimen type.

### UPLC-MS Profiling

5 μl of each extract was injected into the ultra-performance liquid chromatography-mass spectrometry (UPLC-MS) system for metabolomic fingerprinting (Chen et al., 2018; Chen et al., 2020). The injection order of test samples was randomized within placental tissue and cord plasma. The QC sample was injected after every 10 test samples to provide process control. An ACQUITY I Class UPLC system (Waters, Milford, MA, United States), a Waters ACQUITY UPLC HSS T3 column (2.1 × 100 mm, 1.8 μm) and a Waters ACQUITY UPLC HSS T3 VanGuardTM precolumn (2.1 × 5 mm, 1.8 μm) were collectively applied to separate metabolites. A 25.5-min gradient at 450 μl/min was adopted. Formic acid (0.1%) and ammonium formate (5 mmol/L) (pH 9) were utilized as mobile phase A in the positive and negative modes, respectively. Acetonitrile was used as mobile phase B in both modes. All mobile phases were freshly prepared to avoid bacterial contamination. The gradient was set as follows: 0–1 min: 2–5% B; 1–3 min: 5–40% B; 3–17 min: 40–98% B; 17–23 min: 98% B; 23–23.1 min: 98–2% B, 23.1–25.5 min: 2% B. The UPLC system was coupled with a G2-S QTOF system (Waters, Milford, MA, United States) in MSE mode at a resolution of 30,000 and a scan rate of 0.2 s in the mass range from 50 to 2000, with running parameters for both the positive and negative modes as described below: 3 kV capillary voltage, 40 V cone voltage, 80 V source offset, 120°C source temperature, 40°C desolvation temperature, 5 h/L cone gas flow and 800 L/h desolvation gas flow.

### Statistics

For clinical data, normally distributed variables are presented as mean (standard deviation, *SD*) and were analyzed with unpaired Student’s t-test; nonnormally distributed data are presented as median [interquartile range, *IQR*] and were analyzed with Mann-Whitney test; categorical data are described as numbers (%) and were analyzed with Fisher’s exact test. PRISM version 8.0 (GraphPad Software Inc., San Diego, CA, United States) was used, and a significance level of *p-value* < 0.05 was applied.

Metabolic raw data were imported into Progenesis QI™ (Waters, Milford, MA, United States), a software that enabled peak alignment, peak selection, deconvolution, and the annotation of metabolites against the HMDB, ChemSpider, LipidBlast, METLIN, and CCS library databases. Mass error, fragmentation and isotope similarity were scored: maximum score for each criterion was 20; hence, the overall score was 60. Only ions with a mass threshold within ±5 ppm and an overall score ≥36 were considered to be highly confident and retained for further investigation. For ions with more than one possible annotation, the most likely one was manually annotated based on the overall score (higher score preferred) and the origin (endogenous compound preferred). The ions and corresponding peaks were then exported into EZinfo™ (Umetrics, Umeå, Sweden) for a combinational analysis using ANOVA, principal component analysis (PCA) and orthogonal partial least squares-discriminant analysis (OPLS-DA). The scattering plot of PCA was used to evaluate the data quality by checking the clustering pattern of QC samples. The variable importance for projection (VIP) from OPLS-DA was used to gauge the statistical significance of the metabolite features that contributed to the difference between the two groups in each comparison. The criteria for differential metabolites were: 1) VIP >1.0; 2) ANOVA *p-value* < 0.05; and 3) max fold change (FC) > 2.0.

These metabolites were classified by 1) their presence in different comparisons (different regions in Venn diagrams) and 2) corresponding categories in the Human Metabolome Database (HMDB) (in pie charts). The metabolic differences between placental tissue and cord plasma were analyzed by Fisher’s exact test using PRISM version 8.0 (GraphPad Software Inc., San Diego, CA, United States) with the significance level set at a *p-value* < 0.05.

The networks of metabolites, based on their chemical and biochemical relationships, were reconstructed using MetaMapp (Barupal et al., 2012) and plotted by Cytoscape (Version 3.8.0). The Quantitative enrichment analysis (QEA) (Csardi and Nepusz, 2006) of metabolic pathways (referencing KEGG) (Xia and Wishart, 2010) was performed on MetaboAnalyst platform to investigate functional differences within each comparison. Pathways with more than 1 enriched metabolite, QEA *p-value* < 0.05, and false discovery rate (FDR) < 0.05 were recognized as potential pathways of interest.

Multivariable linear regression was performed within each cohort to investigate the correlation between metabolites and fetal growth, adjusted for maternal age and GA. Birthweight and birth length were set as dependent variables separately; the test was repeated for each differential metabolite, which was set as an independent variable, while maternal age and GA were considered confounding factors. Statistics Analysis Software version 9.4 (SAS Institute Inc., Cary, NC, United States) was used, and a *p-value* < 0.05 was applied as the significance level. The statistically significant estimate (β) with its 95% confidence interval (CI) was plotted in forest plots using PRISM version 8.0 (GraphPad Software Inc., San Diego, CA, United States).

## Results

### The Rationale Underlying Our Study Design

This study was designed to evaluate metabolic differences associated with sIUFD in MCDA twin pregnancy by examining placental tissue and cord blood plasma. We considered placental tissue and cord plasma as specimens for the “environment” on the maternal and fetal sides of the interface, respectively. Additionally, to assess the relative impacts of the mode of sIUFD and overall fetal number on the metabolome, we performed four comparisons (Figure 1): 1) In comparison 1 (C1), the surviving cotwins after sIUFD in MCDA twin pregnancies (*n* = 16) were compared to twins from uncomplicated MCDA twin pregnancies (8 pregnancies and 16 neonates); 2) In comparison 2 (C2), the surviving cotwins after sIUFD in MCDA twin pregnancies (*n* = 16) were compared to singletons from uncomplicated singleton pregnancies (*n* = 8); 3) In comparison 3 (C3), comparisons were conducted within the sIUFD group, wherein “spontaneous” sIUFD (*n* = 8) was compared to iatrogenic sIUFD due to single termination a fetus by RFA (*n* = 8); and 4) In comparison 4 (C4), a general comparison of uncomplicated MCDA twin pregnancies (8 pregnancies and 16 neonates) and singleton pregnancies (*n* = 8) was performed.

### Clinical Characteristics of Mothers and Neonates

The demographic and clinical information of the mothers is displayed in Table 1. There were no significant differences in maternal age, smoking or alcohol use, parity, mode of conception, mode of delivery or other obstetric complications (except for PTB). Nevertheless, there was heterogeneity regarding body mass index (BMI); specifically, patients with sIUFD in MCDA twin pregnancies had a smaller increase in BMI (weight) with advancing gestation (C1, mean [5.6 vs. 7.6], *p* < 0.05), and the sIUFD MCDA cohort had a lower maternal BMI, both during the pre-pregnancy stage and at delivery, than the uncomplicated singleton pregnancy cohort (C2, *p* < 0.05). Compared to those of the uncomplicated singleton pregnancies, the rates of PTB in the MCDA cohorts with and without sIUFD were higher (*p* < 0.01), and the gestational ages (GAs) at delivery were lower (*p* < 0.0001) (C1 and C4).

**TABLE 1:** Demographic and clinical characteristics of mothers.

	Groups								
Characteristic	Singleton (*n* = 8)	MCDA (*n* = 8)	sIUFD			*p-value* of Comparisons^a^			
			RFA (*n* = 8)	Spontaneous (*n* = 8)	RFA + Spontaneous (*n* = 16)	C1	C2	C3	C4
Maternal age at delivery (years)^b^	28.5 [25.3–30.0]	29.0 [24.8–32.5]	24.0 [23.3–32.0]	30.0 [27.5–35.0]	29.0 [24.0–32.8]	0.9650	0.8913	0.2030	0.6629
Body mass index, BMI (kg/m^2^)^c^									
BMI before pregnancy	22.6 (3.3)	20.6 (1.6)	19.8 (1.9)	20.6 (2.3)	20.2 (2.1)	0.5974	0.0364*	0.4663	0.1439
BMI at delivery	28.9 (3.2)	28.2 (2.4)	24.9 (2.7)	26.6 (3.5)	25.7 (3.1)	0.0645	0.0323*	0.3215	0.6434
BMI increment	6.3 (2.1)	7.6 (2.1)	5.1 (1.4)	6.0 (2.4)	5.6 (2.0)	0.0316*	0.4308	0.4307	0.2340
Smoking or alcohol use^d^	0	0	0	0	0	>0.9999	>0.9999	>0.9999	>0.9999
Multiparity^d^	7 (88%)	4 (50%)	3 (38%)	4 (50%)	7 (44%)	>0.9999	0.0791	>0.9999	0.2821
Assisted reproduction^d^	0	1 (13%)	0	1 (13%)	1 (6%)	>0.9999	>0.9999	>0.9999	>0.9999
Obstetric complications^d^									
GDM	0	0	3 (38%)	3 (38%)	6 (38%)	0.0664	0.0664	>0.9999	>0.9999
PAS	0	0	1 (13%)	1 (13%)	2 (13%)	0.5362	0.5362	>0.9999	>0.9999
FGR	0	0	0	3 (38%)	3 (19%)	0.5257	0.5257	0.2000	>0.9999
ICP	0	0	0	1 (13%)	1 (6%)	>0.9999	>0.9999	>0.9999	>0.9999
PROM/PPROM	0	1 (13%)	3 (38%)	1 (13%)	4 (25%)	0.6311	0.2622	0.5692	>0.9999
PTB	0	6 (75%)	5 (63%)	7 (88%)	12 (75%)	>0.9999	0.0013**	0.5692	0.0070**
Cesarean delivery^d^	8 (100%)	8 (100%)	4 (50%)	8 (100%)	12 (75%)	0.2622	0.2622	0.0769	>0.9999
Gestational age (weeks)									
Delivery^c^	39.2 (0.3)	36.2 (1.2)	35.4 (3.0)	34.1 (2.2)	34.7 (2.6)	0.1520	<0.0001****	0.3105	<0.0001****
sIUFD occurrence^b^	NA	NA	22.5 [19.0–25.5]	31.0 [30.0–32.0]	28.0 [22.3–31.8]	-	-	0.0120*	-
Interval from sIUFD to delivery^b^	NA	NA	14.8 [6.5–16.1]	1.6 [1.2–5.5]	5.8 [1.6–15.4]	-	-	0.0042**	-

Data are presented as mean (standard deviation, *SD*) if normally distributed data, as median [25th–75th percentile, *IQR*] if not normally distributed data, and as number of mothers (%) if categorical.

a C1: (RFA + Spontaneous) vs. MCDA; C2: (RFA + Spontaneous) vs. Singleton; C3: Spontaneous vs. RFA; C4: MCDA vs. Singleton.

b Mann Whitney test was used.

c Unpaired *t* test was used.

d Fisher’s exact test was used.

*p < 0.05; **p < 0.01; ***p < 0.001; ****p < 0.0001.

Abbreviations: MCDA, monochorionic diamniotic; sIUFD, single intrauterine fetal death; RFA, radiofrequency ablation; GDM, gestational diabetes mellitus; PAS, placenta accreta spectrum; FGR, fetal growth restriction; ICP, intrahepatic cholestasis of pregnancy; PROM, prelabor rupture of membranes; PPROM, preterm prelabor rupture of membranes; PTB, preterm birth; NA, not available.

The bold values are statistically significant *p*-values.

The clinical characteristics of the neonates are shown in Table 2. Relative to those of uncomplicated singleton fetuses, birthweight and birth length were lower among viable cotwins from sIUFD pregnancies (C2) and twins from uncomplicated MCDA twin pregnancies (C4). There was heterogeneity in the Apgar score and neonatal intensive care unit (NICU) admission rate among the individuals in the C1 and C2 comparisons.

**TABLE 2:** Demographic and clinical characteristics of neonates.

	Groups								
Characteristic	Singleton (*n* = 8)	MCDA (*n* = 16, 8 pairs)	sIUFD			*p-value* of Comparisons^a^			
			RFA (*n* = 8)	Spontaneous (*n* = 8)	RFA + Spontaneous (*n* = 16)	C1	C2	C3	C4
Newborn sex^b^						>0.9999	>0.9999	0.3147	>0.9999
Male	4 (50%)	8 (50%)	3 (38%)	6 (75%)	9 (56%)				
Female	4 (50%)	8 (50%)	5 (62%)	2 (25%)	7 (44%)				
Neonatal birthweight (g)^c^	3335.6 (271.4)	2561.9 (316.0)	2362.5 (552.7)	2311.3 (354.5)	2336.9 (449.3)	0.1118	**<0.0001** ^****^	0.8284	**<0.0001** ^****^
Neonatal birth length (cm)^c^	49.4 (1.2)	46.1 (1.9)	45.6 (2.6)	44.5 (4.6)	45.1 (3.7)	0.3424	**0.0040** ^**^	0.5571	**0.0002** ^***^
Apgar score^d^									
1 min	10.0 [10.0–10.0]	10.0 [9.3–10.0]	9.0 [8.3–10.0]	9.0 [7.5–9.0]	9.0 [8.3–9.0]	**0.0020** ^**^	**0.0027** ^**^	0.1501	0.5626
5 min	10.0 [10.0–10.0]	10.0 [10.0–10.0]	10.0 [9.0–10.0]	9.5 [9.5–10.0]	10.0 [9.0–10.0]	**0.0068** ^**^	0.0538	0.7063	>0.9999
10 min	10.0 [10.0–10.0]	10.0 [10.0–10.0]	10.0 [9.3–10.0]	10.0 [9.0–10.0]	10.0 [9.0–10.0]	**0.0434** ^*^	0.1304	>0.9999	>0.9999
NICU admission^b^	0	4 (25%)^e^	4 (50%)	6 (75%)	10 (63%)	0.0732	**0.0064** ^**^	0.6084	0.2622
Etiology of sIUFD^b^									
Cardiac anomalies	NA	NA	4 (50%)	0	4 (25%)	—	—	0.0769	—
TTTS	NA	NA	4 (50%)	1 (13%)	5 (31%)	—	—	0.2821	—
sIUGR	NA	NA	0	6 (74%)	6 (38%)	—	—	**0.0070** ^**^	—
Unexplained death	NA	NA	0	1 (13%)	1 (6%)	—	—	>0.9999	—

Data are presented as mean (standard deviation, *SD*) if normally distributed data, as median [25th–75th percentile, *IQR*] if not normally distributed data, and as number of neonates (%) if categorical.

a C1: (RFA + Spontaneous) vs. MCDA; C2: (RFA + Spontaneous) vs. Singleton; C3: Spontaneous vs. RFA; C4: MCDA vs. Singleton.

b Fisher’s exact test was used.

c Unpaired *t* test was used.

d Mann Whitney test was used.

*p < 0.05; **p < 0.01; ***p < 0.001; ****p < 0.0001.

e Two pairs of twins (four neonates) were admitted to NICU in the MCDA group.

Abbreviations: MCDA, monochorionic diamniotic; sIUFD, single intrauterine fetal death; RFA, radiofrequency ablation; NICU, neonatal intensive care unit; TTTS, twin-twin transfusion syndrome; sIUGR, selective intrauterine growth restriction.

The bold values are statistically significant *p*-values.

As presented in Table 1, although GA at delivery was statistically similar among sIUFD participants (*p* = 0.3105), sIUFD occurred at an earlier GA after RFA (*p* = 0.0120), which consequently prolonged the corresponding GA interval (*p* = 0.0042). A description of the underlying etiologies for sIUFD in MCDA twin pregnancies is provided in Table 2.

### Total Differential Metabolites in Placental Tissue and Cord Plasma

The stability and repeatability of the system are shown in Supplementary Figure S1.

A summary of all metabolites showing differential levels for each of the comparisons undertaken is detailed in Supplementary Table S1. In total, 131 differential metabolites were noted in placental tissue across all comparisons. Classifiable metabolites included 7 (5.34% of 131 total) steroids and steroid derivatives, 34 (25.95%) complex lipids, 22 (16.79%) fatty acyls, 10 (7.63%) organic acids, 4 (3.05%) organonitrogen or organooxygen compounds, 5 (3.82%) benzenoid compounds, and 5 (3.82%) compounds of other categories, while 44 (33.59%) uncharacterized metabolites were also identified. There were 111 differential metabolites identified in cord plasma, comprising complex lipids (12/111 or 10.81%), steroids and steroid derivatives (11/111, 9.91%), fatty acyls and organic acids (both 9/111 or 8.11%), organonitrogen or organooxygen compounds (6/111, 5.41%), and benzenoid compounds (4/111, 3.60%). A total of 13 (11.71%) other compounds and 47 (42.34%) unknown compounds were also identified in cord plasma.

### Metabolic Differences Between Placental Tissue and Cord Plasma

**FIGURE 2:**
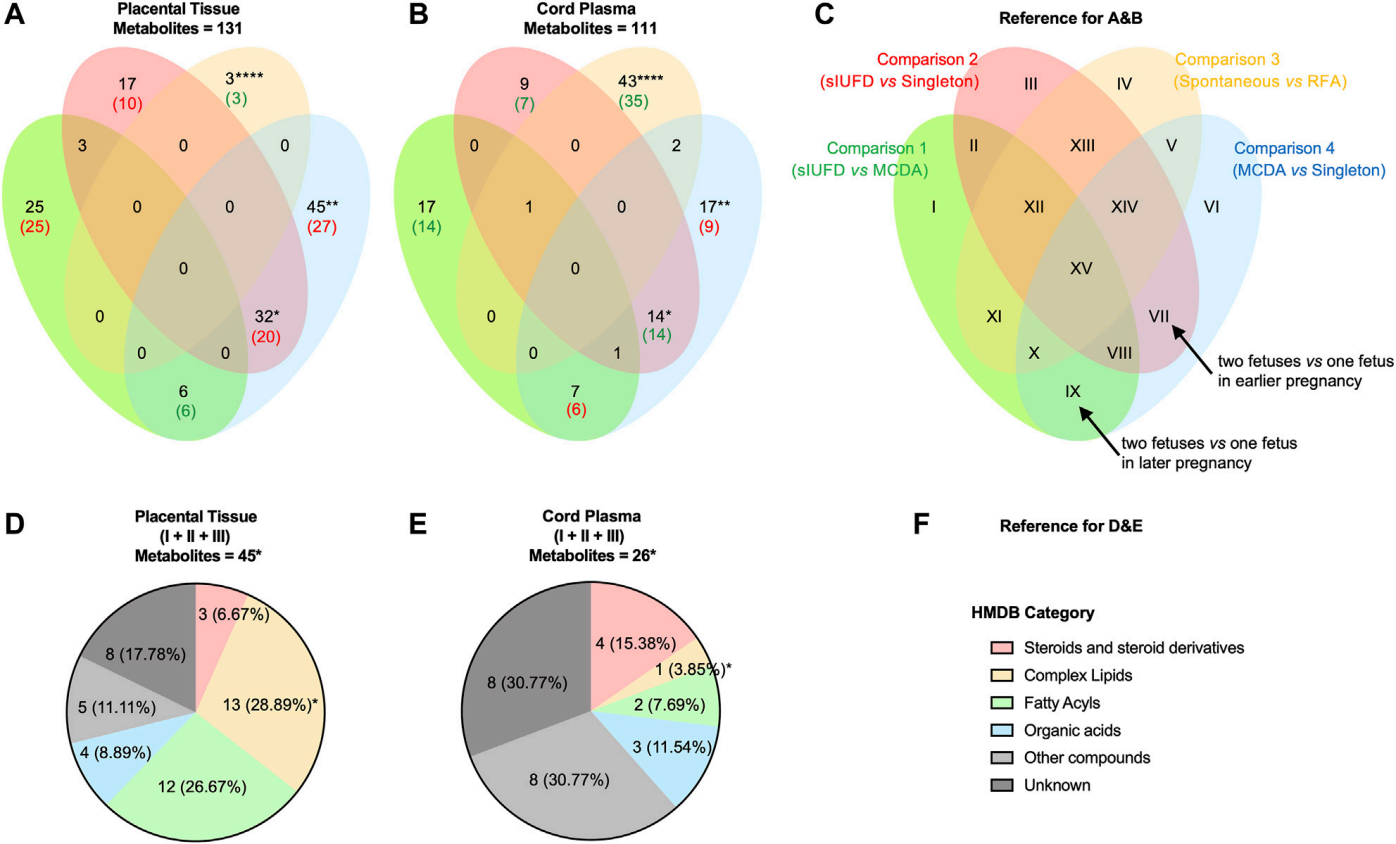
Metabolic differences between placental tissue and cord plasma. Venn diagrams show the overlap in differential metabolites identified from comparisons made in placental tissue **(A)** and in cord plasma **(B)**, and the data represent the number of metabolites: the total number is shown in black; the main expression trend is shown either in red (upregulated) or in green (downregulated). **(C)** The reference for A and B. The four comparisons are represented as four ellipses in their iconic colors, which are C1: in green, (RFA + Spontaneous) vs. MCDA; C2: in red, (RFA + Spontaneous) vs. Singleton; C3: in orange, Spontaneous vs. RFA; and C4: in blue, MCDA vs. Singleton. Thus, differential metabolites may present in 15 regions (from I to XV). To better understand the expression trend, the representative meaning after logical reasoning of two regions are noted (arrow): VII (C4+C2): two fetuses *vs*. one fetus in earlier pregnancy; and IX (C4+C1): two fetuses *vs*. one fetus in later pregnancy. Pie charts illustrate the category (according to the HMDB) of metabolites in region I to region III (combined), in placental tissue **(D)** and in cord plasma **(E)**, and the data represent the number (%) for compounds of each category. **(F)** The reference for D and E. Fisher’s exact test was conducted to analyze the difference between placental tissue and cord plasma. * <0.05; ** <0.01; *** <0.01; **** <0.0001. Abbreviations: MCDA, monochorionic diamniotic; sIUFD, single intrauterine fetal death; RFA, radiofrequency ablation.

For the comparison between two modes of sIUFD [C3 (spontaneous *vs*. RFA), Figures 2A–C (especially region IV)], cord plasma has presented a much larger number of differential metabolites when compared to placental tissue (43/111 vs. 3/131, *p* < 0.0001), and the majority of these metabolites were downregulated on both sides of the interface if sIUFD occurred spontaneously (35/43 and 3/3). In addition, these mode-related metabolites were specific to the plasma network (Supplementary Figure S2 [orange dots]), where lipids were highly involved.

As shown in Figures 2A–C, the metabolic changes associated with carrying two fetuses relative to one fetus, throughout pregnancy (region VI) and in earlier (region VII) or later pregnancy (region IX), can be inferred from C4 (MCDA vs. singleton) with C2 (sIUFD vs. singleton) or C1 (sIUFD vs. MCDA). More species of metabolites were found to be differentially expressed in placental tissue for regions VI (45/131 vs. 17/111, *p* < 0.01) and VII (32/131 vs. 14/111, *p* < 0.05). Additionally, at the maternal side, twin pregnancies had higher metabolite levels during the earlier period (20/32) but lower levels later in pregnancy (6/6), which was opposite to the dynamic for fetal circulation (14/14 and 6/7). In the placental network, where more fetal number-related compounds were identified, fatty acyls (e.g., L-acetylcarnitine), glycerophospholipids or glycerolipids (e.g., PE (20:4 (5Z,8Z,11Z,14Z)/18:1 (11Z))), and sphingolipids (e.g., gangliosides) were noted (Supplementary Figure S2 [red and blue dots]).

Regardless of whether the offspring was full term, the levels of metabolite variations in placental tissue and cord plasma were similar between sIUFD MCDA and uncomplicated MCDA pregnancies (C1, region I) and between sIUFD MCDA and singleton pregnancies (C2, region III) (Figures 2A–C, 25/131 vs. 17/111 and 17/131 vs. 9/111), but in opposite directions (25/25 and 10/17 upregulated vs. 14/17 and 7/9 downregulated). In addition, the comparison of sIUFD pregnancies with all uncomplicated cases (region I to region III combined) revealed more metabolic variation in the placenta (Figures 2D–F, 45/131 vs. 26/111, *p* < 0.05), especially for complex lipids (13/45 vs. 1/26, *p* < 0.05).

Detailed information can be obtained from Supplementary Table S1. No enrichment of any specific metabolic pathway was identified (Supplementary Figure S3).

### Correlation Between Fetal Growth and Metabolites

**FIGURE 3:**
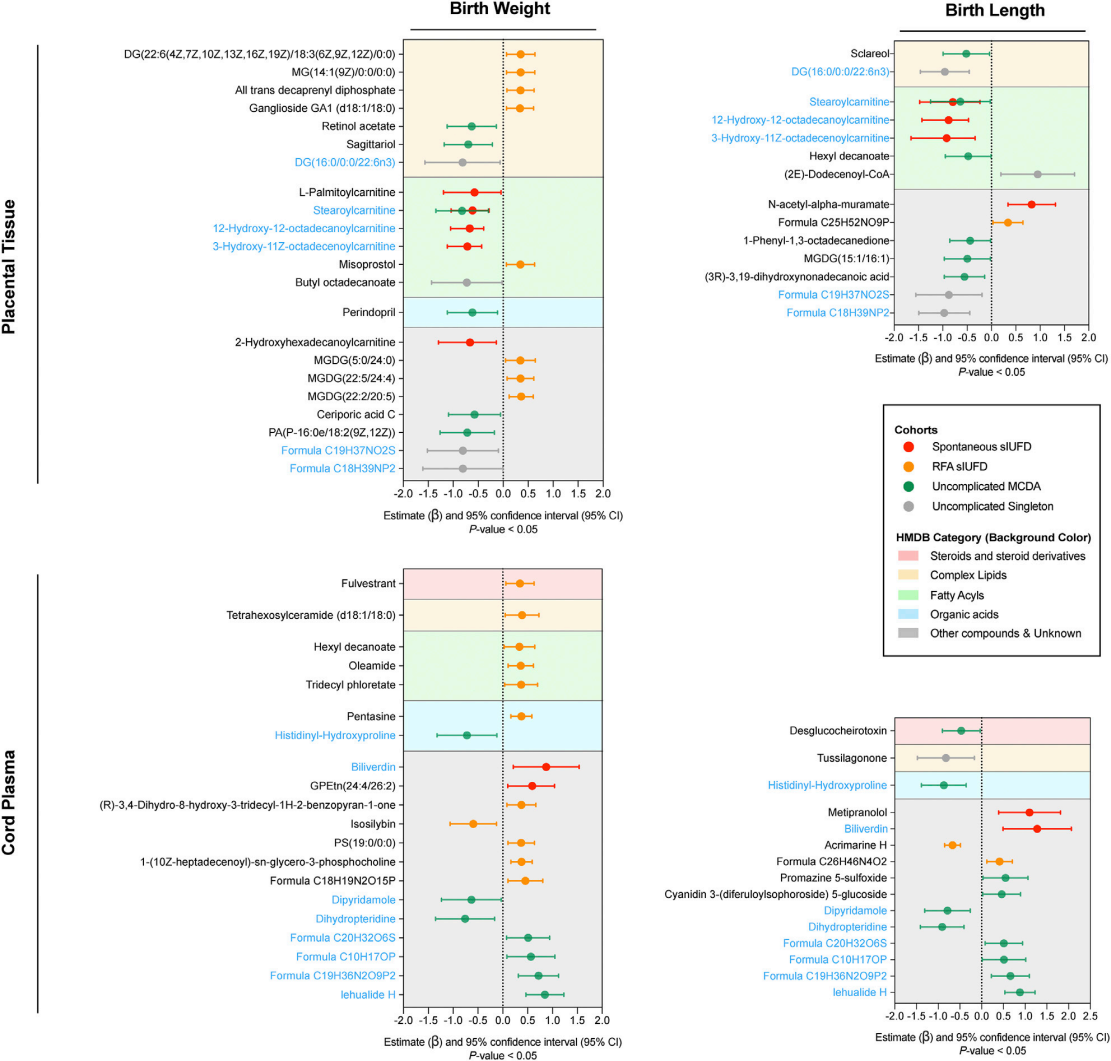
Correlation between fetal growth and metabolites. Only the metabolites that were statistically correlated (*p-value* < 0.05) with neonatal birthweight (left) or birth length (right) are plotted in the forest plots for placental tissue (upper) and cord plasma (lower). In each plot, the colored line represents the estimate (β) and its 95% CI for metabolites among certain groups (noted in the box); the center dotted line indicates a noncorrelation, to the right of which a positive correlation is shown and to the left of which a negative correlation is shown. Metabolites were classified in accordance with their chemical properties (referencing HMDB) and plotted with different background colors; the corresponding category reference is noted in the box. Metabolites that correlated with both birthweight and birth length within either placental tissue or cord plasma are indicated in blue. Abbreviations: sIUFD, single intrauterine fetal death; RFA, radiofrequency ablation; MCDA, monochorionic diamniotic.

In terms of placental tissue, 22 metabolites were significantly associated with neonatal birthweight (Figure 3 [upper left]), most of which were complex lipids (7 [31.82%]) and fatty acyls (6 [27.27%]). In contrast, 14 metabolites were found to be related to birth length (upper right), among which 5 (35.71%) were fatty acyls. Notably, six metabolites were common to both neonatal outcomes. For example, DG (16:0/0:0/22:6n3) in uncomplicated singleton pregnancies, stearoylcarnitine in MCDA twin pregnancies without sIUFD or following spontaneous sIUFD, and two octadecanoyl carnitines in the spontaneous sIUFD group. The majority of these placental metabolites were negatively associated with fetal growth, but those found in the RFA sIUFD group were positively correlated with birthweight.

Regarding metabolites in plasma, 20 metabolites were associated with birthweight, and 15 metabolites were associated with birth length (Figure 3 [lower]); eight of these were associated with both outcomes and showed similar patterns. Histidinyl-hydroxyproline, an amino acid, was negatively associated with fetal growth in uncomplicated MCDA twin pregnancies. Regarding the surviving offspring of sIUFD, many metabolites, including biliverdin, were positively associated with intrauterine growth. Further information about the correlation can be found in Supplementary Table S2.

## Discussion

In the analysis of the surviving twins of sIUFD in MCDA twin pregnancies, metabolic alterations were identified on both the maternal (placental basal plate) and fetal (umbilical cord venous plasma) sides of the interface. Some differential metabolites, including acylcarnitines and gangliosides in the placenta, and histidinyl-hydroxyproline, biliverdin and some xenobiotics in cord blood, were correlated with intrauterine fetal growth (biometry).

Across the four specific comparisons, similar numbers of differential metabolites were identified in placental tissue and cord plasma, but the compositions of these metabolites in the comparisons were distinct according to specimens being tested. The goal of RFA is to stop acute twin-to-twin transfusion when iatrogenic termination of a single fetus occurs in monochorionic twins (Gaerty et al., 2015) in this study, RFA in sIUFD (C3) was associated with many metabolic shifts (mostly upregulations) in the umbilical blood of the surviving twin, representing a possible decrease in metabolite output from the surviving fetus to the non-surviving fetus. This may, however, be confounded by their intrinsic etiologies but is less likely to be affected by the sIUFD delivery intervals, which have no definitive relationship with neonatal prognosis (Saito et al., 1999). Regarding the fetal number, the near doubling of nutrient transportation from the shared placenta (Institute of Medicine, 1990) to fetuses of a MCDA twin pregnancy is a possible explanation for the abundant metabolite stock at that site, especially in earlier pregnancy when the fetal contribution to many metabolic pathways associated with development is negligible (C4 and C2). Importantly, placental metabolites, especially lipids, were more altered with sIUFD (C1 and C2). Lipids were more abundant on the maternal placenta (Cetin et al., 2009), allowing easier detection of the differential lipids in the placental tissue, such as various acylcarnitines and some essential fatty acids (octadecadienoic acid and arachidonic acid) (Supplementary Table S1A).

Lipids are essential for fetal growth (Bobiński and Mikulska, 2015). In this study, three placental acylcarnitines were negatively associated with birthweight and birth length in the spontaneous sIUFD or uncomplicated MCDA group. The underlying mechanism of acylcarnitines in regulating intrauterine human growth remains uncertain (Manta-Vogli et al., 2020), although acylcarnitines are also upregulated in preeclampsia and PTB (Liu et al., 2017; Liu et al., 2020). Gangliosides play positive roles in the development of the fetal brain (Wang et al., 2016). Interestingly, we found a higher placental ganglioside level in the MCDA cohorts relative to singletons (Supplementary Table S1A [C2 and C4]), except for GA1 (d18:1/18:0), which was lower in the sIUFD MCDA group (C2). Our results also suggest a positive correlation between placental GA1 (d18:1/18:0) and birth weight in this RFA-induced sIUFD cohort.

Amino acids (AAs) are key building constituents for fetal and placental development (Vaughan et al., 2017). We detected lower levels of phenylalanine, an essential AA, in the cord plasma of sIUFD offspring (Supplementary Table S1B [C1]). Similar findings have also been seen in both FGR and sIUGR (Bajoria et al., 2001; Paolini et al., 2001), but contradictory findings have also been reported (Wang et al., 2018; Cosmi et al., 2013; Favretto et al., 2012). We also found that histidinyl-hydroxyproline in plasma was higher in twin pregnancies (C4) but lower after sIUFD (C1) and was negatively associated with fetal growth. Additionally, glutathione and glutamic acid accumulated in the placenta of MCDA cases relative to singletons (Supplementary Table S1A [C2 and C4]), while our previous study revealed this highly expressed placental glutamic acid in MCDA twin pregnancies with sIUGR compared to uncomplicated pregnancies (Wang et al., 2018).

Bilirubin in fetal circulation was found to decrease with the time spent carrying two fetuses in the following order: singleton pregnancy > sIUFD in MCDA > uncomplicated MCDA (Supplementary Table S1B). We also found a lower umbilical biliverdin level in the spontaneous sIUFD group than the RFA-induced sIUFD group (C3), and this metabolite is positively associated with antenatal development of the fetus in spontaneous sIUFD. As degradation products of heme, bilirubin and biliverdin have not been investigated in terms of their functions in fetal development, but they are probably related to insufficient and unbalanced blood flow in MC twins. Additionally, the pathophysiology of multiorgan damage on the surviving MC cotwins may contribute and includes the following (Morris et al., 2020): 1) the disseminated intravascular coagulation caused by thromboplastic materials from the retained dead fetus or 2) acute transfusion from the survivor to the demised cotwin.

Some xenobiotics, such as 3-hydroxylidocaine, fulvestrant, and dipyridamole, were also identified in cord plasma, which indicates that xenobiotics taken up by the mother could readily transfer into the fetal circulation, despite the placental barrier (Myllynen et al., 2005). Dipyridamole is used with low-dose aspirin in high-risk pregnancies to prevent idiopathic uteroplacental insufficiency and fetal growth retardation (Uzan et al., 1989). Surprisingly, fetal plasma dipyridamole correlated negatively with intrauterine growth in the uncomplicated MCDA group.

From endogenous metabolic aspects, we found nearly opposite trends in metabolic changes on the two sides of the interface. Specifically, lipids (e.g., gangliosides) and AAs are elevated in the placenta but lower in the cord blood of MCDA and sIUFD pregnancies. Considering the positive effects of these compounds on fetal growth (Wang et al., 2016), the importance of corresponding placental transport system (Mitchell et al., 2012; Lin et al., 2014; Vaughan et al., 2017), and their inferior neonatal outcomes, it is plausible that impaired placental transport of metabolites was involved in the mechanisms of MCDA twin pregnancies with and without sIUFD, and this deserves more study. Similarly, the pharmacological mechanism of some exogenous drugs, such as dipyridamole, and their safety also require further investigation.

Intrauterine development is critical for the lifelong health of an individual (Moisiadis and Matthews, 2014). Differential metabolites positively associated with birthweight and birth length provide possible therapeutic targets for rescuing fetal development of the surviving cotwins following sIUFD, such as prenatal ganglioside supplementation (Ryan et al., 2013; Wang et al., 2020).

In this study, the relative impact of fetal number and mode of sIUFD was considered to further discriminate sIUFD. Additionally, comparisons between the two sides of the maternal-fetal interface were conducted. However, this preliminary comparison may be accurate only when the efficiencies for metabolite extraction and LC-MS profiling are constant in different sample types, which can be further validated by targeted methods.

The chromatographic column is relatively stable in batches and among cohorts (because of the randomized sequence), which is supported by the system pressure (Supplementary Figure S1B). However, there still seemed to be imperfects in the clustering of plasma QCs and two spontaneous sIUFD samples (non-outliers), when normalized with total intensity (Supplementary Figure S1A). QC-based normalization gave slightly different clustering patterns but did not significantly change the list of differential metabolites. Thus, related data are provided as thorough as possible for referring, and results may be applied more critically considering this limitation.

Our study has some other limitations, including the small sample size and strict criteria used to define differential levels of metabolites, that potentially limit the sensitivity of the study, despite increasing specificity. Furthermore, our study did not consider dichorionic or dizygous twin pregnancies or fetal order, limiting the interpretation of the findings (Hillman et al., 2011; Ward et al., 2018). Finally, birthweight and birth length are static indexes (proxies) for fetal growth and are crude. The association with growth rate (Hediger et al., 2005) and postpartum neurodevelopment (Hillman et al., 2011) deserves further consideration.

## Conclusion

This work provides the first evidence that metabolomes in placental tissue and umbilical cord plasma are associated with sIUFD, with each tissue demonstrating specific variations. Although the mechanisms of differential metabolite levels on fetal growth require further clarification, placental transport of lipids and AAs may be involved. Thus, this study presents certain targets that may be suitable for rescuing fetal development.

## Data Availability

The original contributions presented in the study are included in the article/Supplementary Material, further inquiries can be directed to the corresponding authors.

## Abbreviations

ART, assisted reproductive technology; MC, monochorionic; sIUFD, single intrauterine fetal death; RFA, radiofrequency ablation; PTB, preterm birth; MCDA, monochorionic diamniotic; QC, quality control; UPLC-MS, ultra-performance liquid chromatography-mass spectrometry; VIP, variable importance for projection; OPLS-DA, orthogonal partial least squares-discriminant analysis; FC, fold change; HMDB, human metabolome database; CI, confidence interval; BMI, body mass index; GAs, gestational ages; NICU, neonatal intensive care unit; AAs, amino acids.
