# Supplementary figures and images for: The Metabolic Signatures of Surviving Cotwins in Cases of Single Intrauterine Fetal Death During Monochorionic Diamniotic Pregnancy: A Prospective Case-Control Study
Source: Front Mol Biosci. 2022 Apr 8;9:799902. doi: 10.3389/fmolb.2022.799902 (PMC9024353; doi:10.3389/fmolb.2022.799902)

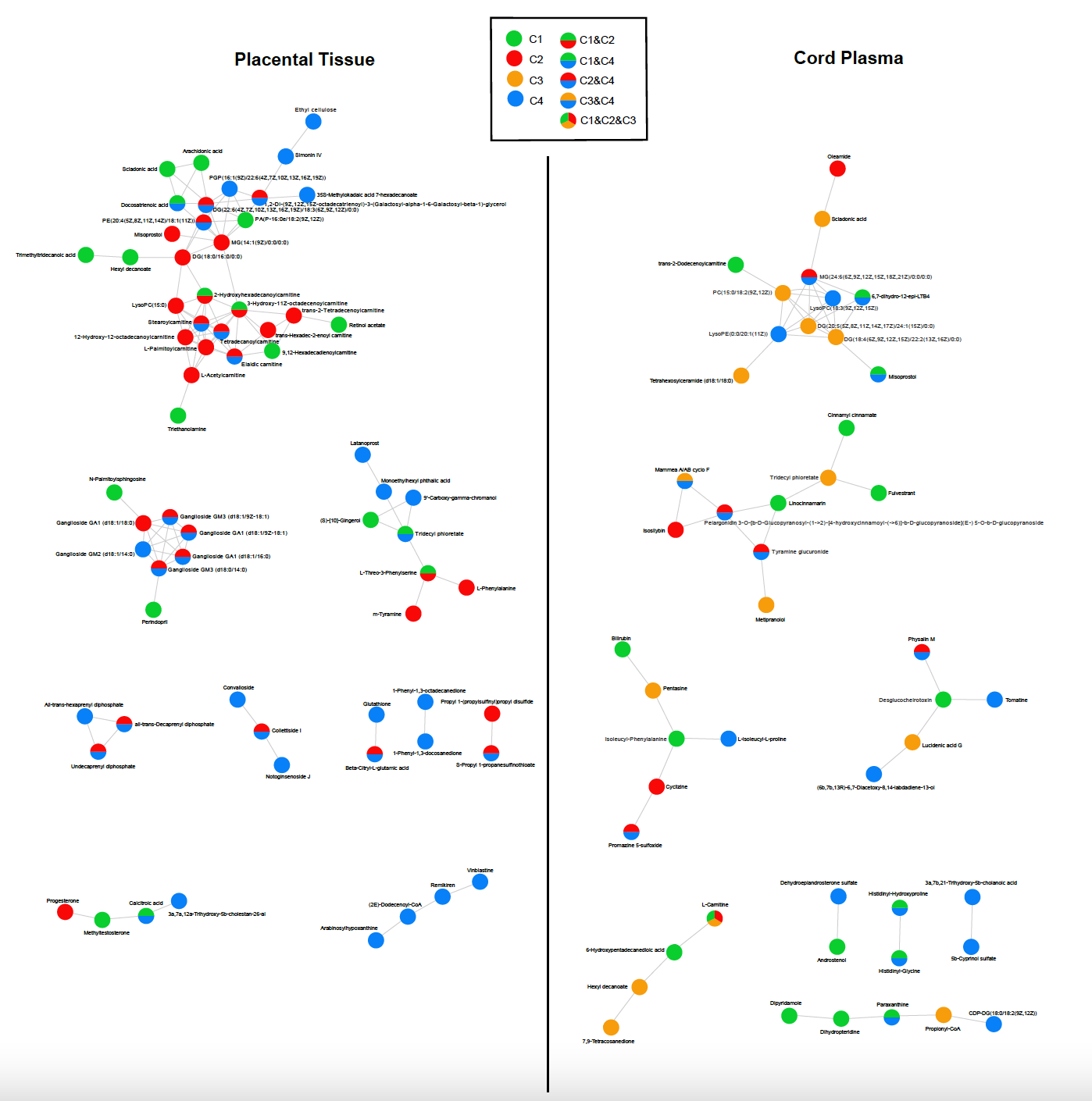

Supplement: Supplementary file 2 [file Image2.PNG]

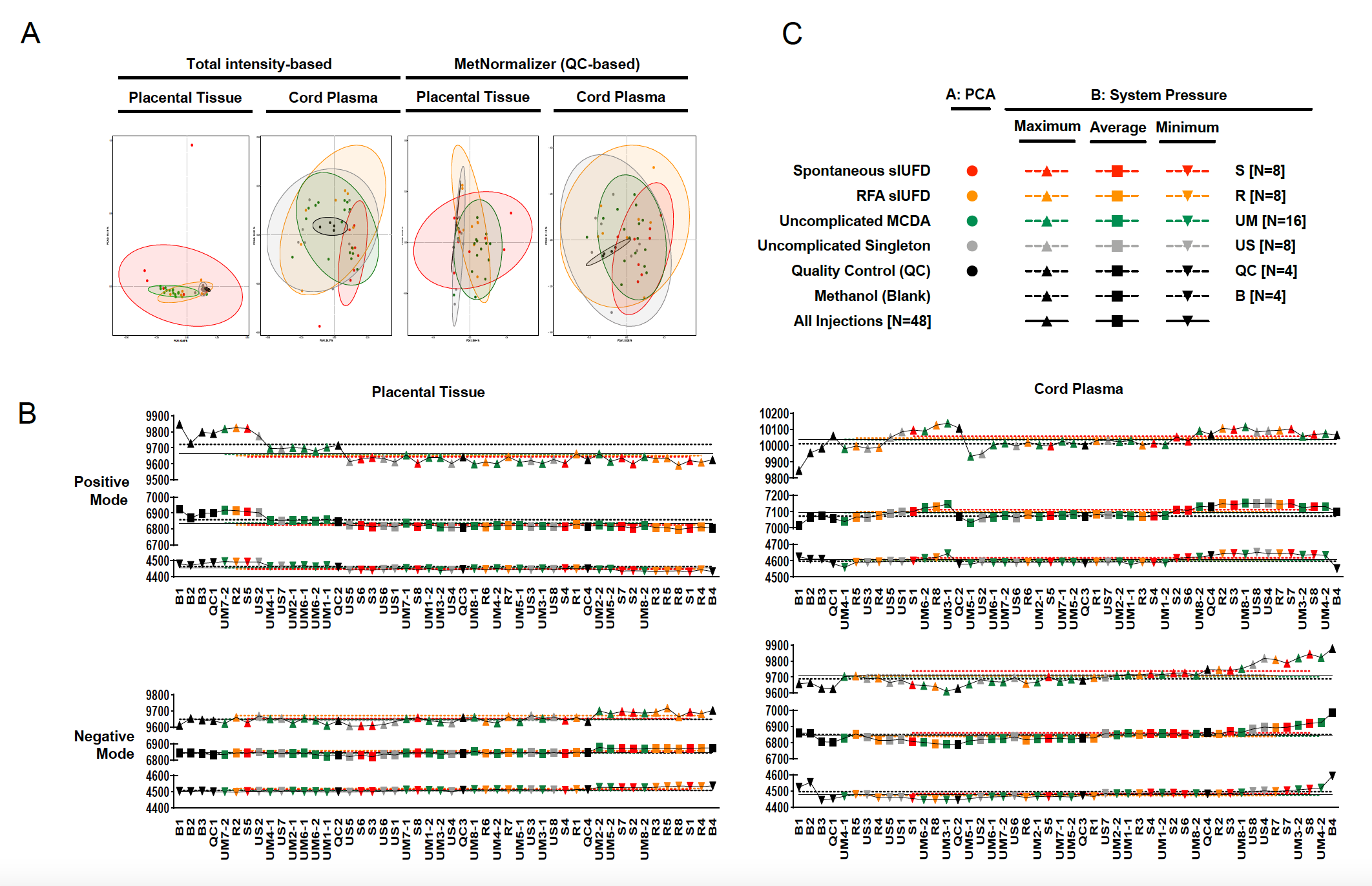

Supplement: Supplementary file 4 [file Image1.PNG]

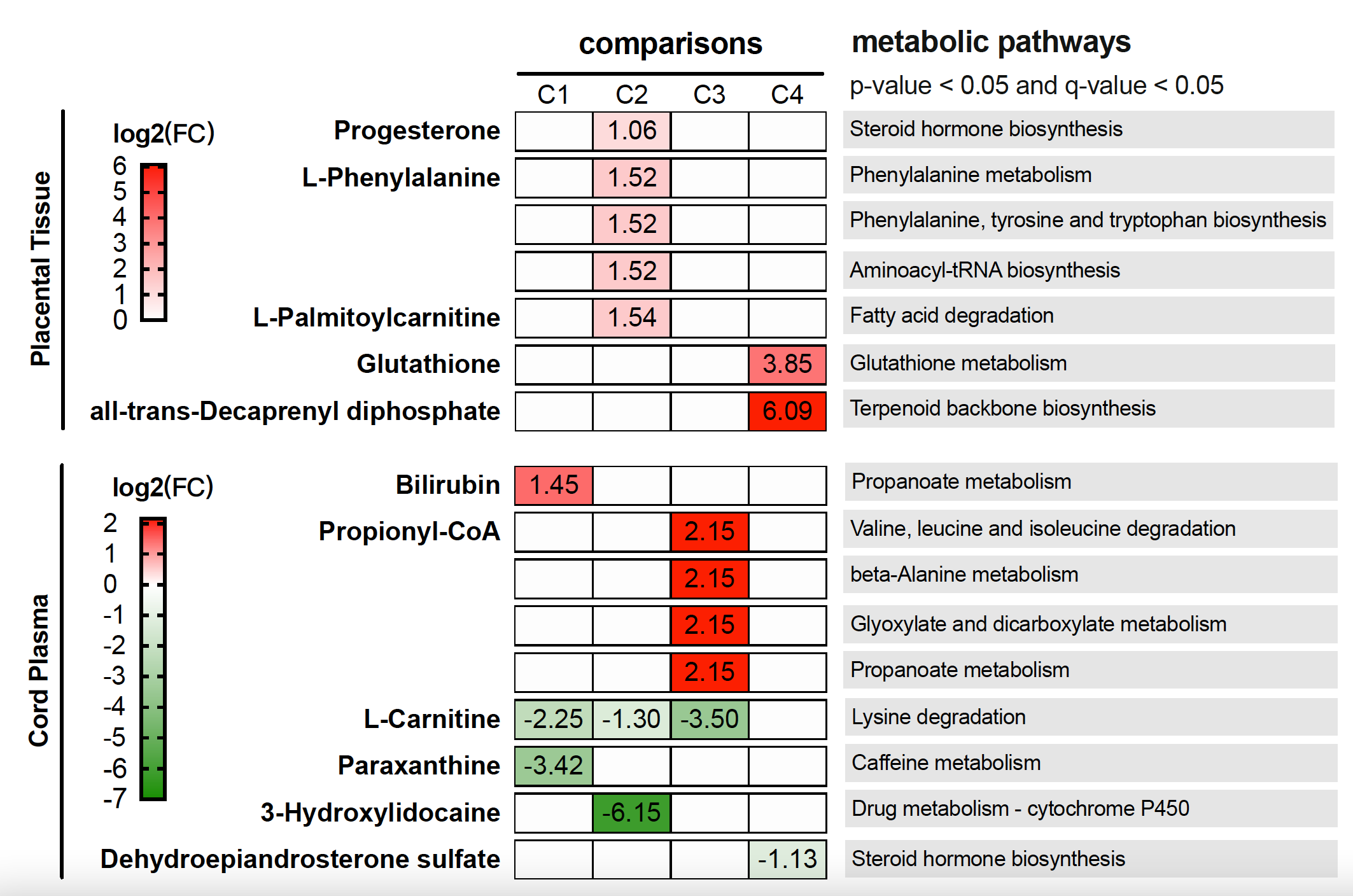

Supplement: Supplementary file 5 [file Image3.PNG]
