# Supplementary material for: The Metabolic Signatures of Surviving Cotwins in Cases of Single Intrauterine Fetal Death During Monochorionic Diamniotic Pregnancy: A Prospective Case-Control Study
Source: Front Mol Biosci. 2022 Apr 8;9:799902. doi: 10.3389/fmolb.2022.799902 (PMC9024353; doi:10.3389/fmolb.2022.799902)
Supplement: Supplementary file 6 [file DataSheet1.docx]

**SUPPLEMENTARY FIGURE LEGENDS**

**Supplementary Figure 1.** The stability and repeatability of the system.

(A) Metabolic raw data from UPLC-MS were normalized based on the total intensity (using Progenesis QI) or based on QC samples (with the R package “MetNormalizer”). Corresponding principal component analysis (PCA) were conducted for placental tissue and cord plasma: 40 test samples (in 4 groups) and 4 quality control (QC) samples were plotted in round symbols with representative group colors. The parameter R2 of QCs, from left to right, are 0.439, 0.492, 0.397 and 0.464, respectively. From total intensity-based normalization, one sample in placenta tissue and another (of a different patient) in cord plasma are outside the corresponding ellipses of the spontaneous sIUFD cohort, but they are not outliers according to the Hotelling's T2 test (via software SIMCA). Data after intensity-based normalization were used for further analysis. (B) The Water Acquity SDS Posturn Reports were reviewed for better understanding the stability of the UPLC system. According to the actual injection order, the maximum, average and minimum system pressures were plotted in different symbols with representative group colors. (C) The symbol reference for A and B. The straight lines represent the average figures from the first till the last injection in corresponding groups (dotted lines), or for all 48 injections (solid line). Abbreviations: sIUFD, single intrauterine fetal death; RFA, radiofrequency ablation; MCDA, monochorionic diamniotic.

**Supplementary Figure 2.** Metabolic networks of differential metabolites.

Two-dimensional networks were constructed based on the chemical or biochemical relationships of metabolites for placental tissue (left) and for cord plasma (right). Metabolites identified from different comparisons were plotted as dots in iconic colors: green for C1, red for C2, orange for C3, and blue for C4. Metabolites presented in multiple comparisons were merged with multiple colors. The four comparisons are C1: (RFA + Spontaneous) *vs* MCDA; C2: (RFA + Spontaneous) *vs* Singleton; C3: Spontaneous *vs* RFA; C4: MCDA *vs* Singleton. Abbreviations: MCDA, monochorionic diamniotic; RFA, radiofrequency ablation.

**Supplementary Figure 3.** Metabolic pathways of differential metabolites.

Heatmaps demonstrate the differences in metabolites and related metabolic pathways within the four comparisons in placental tissue (upper) and in cord plasma (lower). Each column represents each comparison, and each row represents a metabolite (left) with the corresponding pathway (right). The color, with the color key on the left, indicates the relative concentration of metabolite via a log2 scale. The four comparisons are C1: (RFA + Spontaneous) *vs* MCDA; C2: (RFA + Spontaneous) *vs* Singleton; C3: Spontaneous *vs* RFA; and C4: MCDA *vs* Singleton. Pathways with QEA *P-value* < 0.05 and FDR < 0.05 are displayed. Abbreviations: QEA, quantitative enrichment analysis; FDR, false discovery rate; MCDA, monochorionic diamniotic; RFA, radiofrequency ablation.
